# Risk factors and clinical outcomes of cytomegalovirus infection following haploidentical hematopoietic stem cell transplantation in patients with aplastic anemia: a single-center retrospective study

**DOI:** 10.3389/fmed.2024.1523909

**Published:** 2025-01-29

**Authors:** Jia Feng, Xinhe Zhang, Zhengwei Tan, Yuechao Zhao, Huijin Hu, Junfa Chen, Liqiang Wu, Qinghong Yu, Dijiong Wu, Baodong Ye, Wenbin Liu

**Affiliations:** ^1^The First Affiliated Hospital of Zhejiang Chinese Medical University (Zhejiang Provincial Hospital of Traditional Chinese Medicine), Hangzhou, China; ^2^The First School of Clinical Medicine, Zhejiang University of Traditional Chinese Medicine, Hangzhou, China

**Keywords:** Keyword: cytomegalovirus infection, aplastic anemia, haploidentical hematopoietic stem cell transplantation, chronic graft-versus-host disease, immunology

## Abstract

**Background:**

Cytomegalovirus (CMV) infection remains a critical cause of mortality after allogeneic hematopoietic stem cell transplantation, despite significant advancements in CMV prevention and treatment with the introduction and widespread use of letermovir. However, in China, due to limitations in the availability and cost of medications, some patients still face challenges in accessing letermovir. For this subset of the population, exploring the risk factors for CMV infection remains significant in predicting its occurrence.

**Methods:**

Therefore, a retrospective analysis was conducted on 88 haploidentical hematopoietic stem cell transplant recipients over 4 years.

**Results:**

Our study results indicate that chronic graft-versus-host disease (cGVHD) is an independent risk factor for CMV infection following haploidentical hematopoietic stem cell transplantation (Haplo-HSCT). Survival analysis reveals lower survival rates in the refractory CMV infection (RCI) group compared to the non-RCI group, with patients having lower viral loads demonstrating higher rates of seroconversion and improved survival under the same treatment regimen.

**Conclusion:**

Strengthening the monitoring of CMV-DNA in post-transplant patients, actively promoting hematopoietic recovery, preventing the occurrence of CMV infection, and controlling the development of CMV infection can lead to better survival outcomes for patients with aplastic anemia undergoing Haplo-HSCT.

## 1 Introduction

Cytomegalovirus (CMV) is a common and crucial viral infection following allogeneic hematopoietic stem cell transplantation (allo-HSCT) (1). Depending on the type of transplantation and geographical region, the incidence of CMV infection ranges from approximately 40%–70% (2, 3), significantly impacting both the survival and quality of life of affected patients. Despite significant advancements in CMV prevention and treatment with the introduction and widespread use of letermovir, in China, some patients still face challenges in accessing letermovir due to limitations in the availability and cost of medications. For these individuals, CMV infection remains associated with an increased risk of mortality (4), particularly in the case of refractory CMV infection (RCI) (4, 5), which can result in a mortality rate exceeding 80% (6), with CMV pneumonia being the most lethal manifestation. Additionally, the Chinese population resides in a high-risk zone for CMV infection, with an adult CMV serum positivity rate ranging from 80% to 93.7% (7, 8). This significantly increases the likelihood of CMV infection (9). For this subset of the population, exploring the risk factors for CMV infection remains meaningful in predicting its occurrence.

In the current study, we conducted a retrospective analysis of clinical data from 88 patients with aplastic anemia (AA) who underwent haploidentical hematopoietic stem cell transplantation (Haplo-HSCT). The aim was to investigate the incidence of CMV infection and its associated risk factors in haploidentical transplant recipients.

## 2 Materials and methods

### 2.1 Patients

In this study, we retrospectively analyzed the clinical data of 88 patients diagnosed with AA who underwent Haplo-HSCT at the Department of Hematology, Zhejiang Provincial Hospital of Traditional Chinese Medicine, from September 2018 to November 2022. The patients were actively followed up until July 2023. Among the 88 patients (44 males, 44 females), with a median age of 32 years (range: 9–55), there were 70 cases of severe aplastic anemia (SAA) and 18 cases of non-severe aplastic anemia (NSAA), all meeting the diagnostic criteria for AA (10). This study has been approved by the Institutional Review Board of the hospital. The baseline characteristics of the patients are presented in Table 1.

**TABLE 1:** Patient and transplant characteristics according to post-transplant CMV infection.

Factors	*N* (%)	No CMV infection (*n*, %)	CMV infection (*n*, %)	Statistics	*p*-Value
Total	88 (100)	52 (59.1)	36 (40.9)		-
**Patient age at transplantation**					
≤40 years	65 (73.9)	25 (69.4)	40 (76.9)	χ^2^ = 0.616	0.432
40 years	23 (26.1)	11 (30.6)	12 (23.1)		
**Sex**					
Male	44 (50.0)	16 (44.4)	28 (53.8)	χ^2^ = 0.752	0.386
Female	44 (50.0)	20 (55.6)	24 (46.2)		
**Donor sex**					
Male	50 (56.8)	20 (55.6)	30 (57.7)	χ^2^ = 0.040	0.842
Female	38 (43.2)	16 (44.4)	22 (42.3)		
**Donor/recipient sex combination**					
Female to male	18 (20.5)	5 (13.9)	13 (25.0)	χ^2^ = 1.614	0.204
Others	70 (79.5)	31 (86.1)	39 (75.0)		
**Diagnosis**					
NSAA	18 (20.5)	7 (19.4)	11 (21.2)	χ^2^ = 0.038	0.845
SAA	70 (79.5)	29 (80.6)	41 (78.8)		
**The blood type of the donor and the recipient**					
Incompatible	34 (38.6)	12 (33.3)	22 (42.3)	χ^2^ = 0.723	0.395
Compatible	54 (61.4)	24 (66.7)	30 (57.7)		
**Stem cell source**					
PBSCs	10 (11.4)	5 (13.9)	5 (9.6)	χ^2^ = 0.386	0.535
PBSCs + BM	78 (88.6)	31 (86.1)	47 (90.4)		
**Conditioning regimen**					
Bu + Cy	22 (25.0)	7 (19.4)	15 (28.8)	χ^2^ = 1.003	0.317
Flu + Cy + ATG	66 (75.0)	29 (80.6)	37 (71.2)		
**UC-BSC assisted reinfusion**					
No	40 (45.5)	18 (50.0)	22 (42.3)	χ^2^ = 0.508	0.476
Yes	48 (54.5)	18 (50.0)	30 (57.7)		
**MSC assisted reinfusion**					
No	38 (43.2)	15 (41.7)	23 (44.2)	χ^2^ = 0.057	0.811
Yes	50 (56.8)	21 (58.3)	29 (55.8)		
**NE 28-day engraftment**					
No	6 (6.8)	5 (13.9)	1 (1.9)	χ^2^ = 4.740	0.004
Yes	82 (93.2)	31 (86.1)	51 (98.1)		
**PLT 28-day engraftment**					
No	22 (25.0)	7 (19.4)	15 (28.8)	χ^2^ = 1.003	0.317
Yes	66 (75.0)	29 (80.6)	37 (71.2)		
**aGVHD**					
No	48 (54.5)	21 (58.3)	27 (51.9)	χ^2^ = 0.353	0.553
Yes	40 (45.5)	15 (41.7)	25 (48.1)		
**cGVHD**					
No	62 (70.5)	31 (86.1)	31 (59.6)	χ^2^ = 7.174	0.007
Yes	26 (29.5)	5 (13.9)	21 (40.4)		
**EBV infection**					
No	33 (37.5)	18 (50.0)	15 (28.8)	χ^2^ = 4.062	0.044
Yes	55 (62.5)	18 (50.0)	37 (71.2)		

SAA, severe aplastic anemia; NSAA, non-severe aplastic anemia; Bu, busulfan; Cy, cyclophosphamide; FCA, fludarabine, cyclophosphamide, antithymocyte globulin; NE, neutrophils; PLT, platelet; aGVHD, acute graft-versus-host disease; cGVHD, chronic graft-versus-host; EBV, Epstein-Barr virus.

All patients underwent myeloablative conditioning, with 22 patients receiving the BUCY (busulfan/cyclophosphamide) conditioning regimen, and 66 patients undergoing the FCA (fludarabine/cyclophosphamide/antithymocyte globulin) conditioning regimen. A combination of antithymocyte globulin (ATG), mycophenolate mofetil (MMF), cyclosporine (CSA), and short-term methotrexate (MTX) was employed for graft-versus-host disease (GVHD) prophylaxis in all patients. Acute GVHD (aGVHD) and chronic GVHD (cGVHD) were diagnosed according to standard references (11–13). For patients experiencing aGVHD, immediate first-line treatment involved administering methylprednisolone at a dose of 1–2 mg⋅kg^1^⋅d^1^. In cases where methylprednisolone was ineffective or dependency occurred, second-line therapies such as ruxolitinib, anti-CD25 monoclonal antibodies, MMF, among others, were administered. The primary treatment for cGVHD involved the use of methylprednisolone and/or CSA as the first-line approach.

Follow-up for all 88 patients was conducted through methods such as phone interviews and hospital registration systems, with the follow-up deadline set at July 2023. Neutrophil engraftment was defined as a consecutive 3-day absolute neutrophil count (ANC) > 0.5 × 10^9^/L, while platelet engraftment was defined as a consecutive 7-day platelet count (PLT) > 20 × 10^9^/L without requiring platelet transfusions. Primary graft failure was defined according to established literature (14). Overall survival (OS) time post-transplantation was defined as the period from transplantation to either patient death or the last follow-up date.

### 2.2 CMV monitoring, definitions, and antiviral therapy

According to our internal standards, blood CMV-DNA positivity is defined as a quantitative PCR result with a CMV viral load > 1 × 10^2^ copies/ml (15). CMV viremia is defined as two consecutive CMV-DNA tests showing levels exceeding 500 copies/ml, or a single CMV-DNA test result exceeding 1,000 copies/ml (16). In this study, the occurrence of viremia in patients was considered a confirmed CMV infection. RCI is defined as a situation where, after receiving reasonable anti-CMV treatment for 2 weeks, the CMV viral load remains unchanged or increases (17, 18). The definition of CMV-related diseases follows the literature reference (15), while the definitions of Epstein-Barr virus (EBV) viremia and related diseases adhere to the literature reference (19). Prophylaxis for CMV infection with ganciclovir was administered from day 9 to 2 during the pre-transplant period, and acyclovir prophylaxis for herpes virus infection was given from day 1 to 1-year post-transplant.

All patients underwent quantitative PCR monitoring of peripheral blood CMV-DNA and EBV-DNA twice a week from the initiation of pre-transplant conditioning until day +90. From day +90 onward, monitoring was conducted every 1–2 weeks until day +180. After day +180, in the presence of symptoms suggestive of a possible viral infection, simultaneous retesting of CMV-DNA and EBV-DNA was performed. If positivity occurred during this period, the monitoring frequency increased to twice a week until viral clearance. The first-line treatment options for CMV infection included either ganciclovir or sodium phosphonoformate. For RCI, drugs not used in the first-line regimen were selected for monotherapy or combination therapy. Once CMV-DNA became negative for two consecutive tests, acyclovir was administered orally for prophylaxis.

### 2.3 Statistical analyses

Inter-group continuous variables were subjected to two-tailed *t*-tests or Kruskal–Wallis tests, while categorical variables were analyzed using Chi-square tests or Fisher’s exact tests. Logistic regression models for binary variables were employed for both univariate and multivariate analyses, with the latter incorporating all factors from the univariate analysis with a *p*-value < 0.10. The cumulative incidence of CMV infection was computed using a competing risk model. Kaplan–Meier methodology was employed to determine the probability of OS, and comparisons were made using the log-rank test. A *p*-value < 0.05 was considered statistically significant. All statistical analyses were conducted using SPSS 26.0 software, and graphical representations were created using GraphPad (Supplementary Table 1).

## 3 Results

### 3.1 Patient clinical characteristics and hematopoietic recovery

This study included a total of 88 patients with AA who underwent haploidentical transplantation, comprising 44 males and 44 females. The median age at the time of transplantation was 32 years (range: 9–55). Disease classification was as follows: NSAA in 18 cases, SAA in 65 cases, and very severe aplastic anemia (VSAA) in 5 cases. The median time for neutrophil engraftment in 85 patients was 12 days (range: 9–48), with 82 achieving engraftment within 28 days. For platelet engraftment, the median time for 81 patients was 16 days (range: 7–92), with 15 achieving engraftment within 28 days. Ultimately, hematopoietic recovery was achieved in 81 patients, while the remaining 7 patients experienced graft failure, adverse events, or early mortality (Table 1).

### 3.2 Overview of CMV infection, treatment, and outcome

Before transplantation, both donor and recipient CMV-DNA quantification levels were below the detection range (<1 × 10^2^ copies/ml). Among the patients, 70 were CMV-IgG positive, and the remaining 18 were not assessed. By the end of the follow-up period, CMV infection occurred in 52 out of the 88 patients (59.1%). The median time to the first occurrence of CMV infection was 36.5 days (range: 11–189).

After the first-line treatment, 40 patients (76.9%) achieved CMV-DNA negativity, while the remaining patients experienced RCI. Among the 12 RCI patients, 5 (41.7%) achieved viral clearance after receiving second-line treatment, while 6 died with persistent CMV-DNA positivity. The overall rate of viral clearance after CMV infection treatment was 86.5% (45/52).

Among CMV-infected patients, there were 29 cases in the group with the highest viral load below 1 × 10^4^ copies/ml, and the clearance rate was 96.6% (28/29). In the group with a viral load exceeding 1 × 10^4^ copies/ml, there were 23 cases, and the clearance rate was 73.9% (17/23). The difference in clearance outcomes between the two groups was statistically significant (*p* = 0.035).

### 3.3 Risk factors for CMV infection

The results are shown in Table 2. Univariate analysis indicated that neutrophil engraftment beyond 28 days (*p* = 0.004), cGVHD (*p* = 0.007), and EBV infection (*p* = 0.044) were clinical risk factors for CMV infection in AA patients undergoing Haplo-HSCT. Multivariate analysis further identified cGVHD (*p* = 0.043) as an independent risk factor for the occurrence of CMV infection after Haplo-HSCT.

**TABLE 2:** Analysis of risk factors associated with CMV infection after allogeneic hematopoietic stem cell transplantation.

Factors	CMV infection (*n*, %)	Univariate analysis		Multivariate analysis	
		***p*-Value**	**OR value (95% CI)**	***p*-Value**	**OR value (95% CI)**
**Patient age at transplantation**					
≤40 years	40 (76.9)	0.432	0.682 (0.261∼1.778)	–	–
40 years	12 (23.1)				
**Sex**					
Male	28 (53.8)	0.386	0.686 (0.292∼1.611)	–	–
Female	24 (46.2)				
**Donor sex**					
Male	30 (57.7)	0.842	0.917 (0.389∼2.160)	–	–
Female	22 (42.3)				
**Donor/recipient sex combination**					
Female to male	13 (25.0)	0.204	0.484 (0.156∼1.504)	–	–
Others	39 (75.0)				
**Diagnosis**					
NSAA	11 (21.2)	0.845	0.900 (0.312∼2.598)	–	–
SAA	41 (78.8)				
**The blood type of the donor and the recipient**					
Incompatible	22 (42.3)	0.395	0.682 (0.281∼1.652)	–	–
Compatible	30 (57.7)				
**Stem cell source**					
PBSCs	5 (9.6)	0.535	1.516 (0.405∼5.675)	–	–
PBSCs + BM	47 (90.4)				
**Conditioning regimen**					
Bu + Cy	15 (28.8)	0.317	0.595 (0.215∼1.652)	–	–
Flu + Cy + ATG	37 (71.2)				
**UC-BSC assisted reinfusion**					
No	22 (42.3)	0.476	1.364 (0.580∼3.204)	–	–
Yes	30 (57.7)				
**MSC assisted reinfusion**					
No	23 (44.2)	0.811	0.901 (0.381∼2.127)	–	–
Yes	29 (55.8)				
**NE 28-day engraftment**					
No	1 (1.9)	0.004	8.226 (0.918∼73.716)	0.169	4.831 (0.513∼45.498)
Yes	51 (98.1)				
**PLT 28-day engraftment**					
No	15 (28.8)	0.317	0.595 (0.215∼1.652)	–	–
Yes	37 (71.2)				
**aGVHD**					
No	27 (51.9)	0.553	1.296 (0.550∼3.055)	–	–
Yes	25 (48.1)				
**cGVHD**					
No	31 (59.6)	0.007	4.200 (1.405∼12.555)	0.043	3.244 (1.035∼10.042)
Yes	21 (40.4)				
**EBV infection**					
No	15 (28.8)	0.044	2.467 (1.0126∼5.989)	0.341	1.597 (0.609∼4.185)
Yes	37 (71.2)				

SAA, severe aplastic anemia; NSAA, non-severe aplastic anemia; Bu, busulfan; Cy, cyclophosphamide; FCA, fludarabine, cyclophosphamide, antithymocyte globulin; NE, neutrophils; PLT, platelet; aGVHD, acute graft-versus-host disease; cGVHD, chronic graft-versus-host; EBV, Epstein-Barr virus; CMV, cytomegalovirus; –, multivariate analyses were not included.

### 3.4 Overall prognosis and survival analysis of patients with CMV infection

**FIGURE 1:**
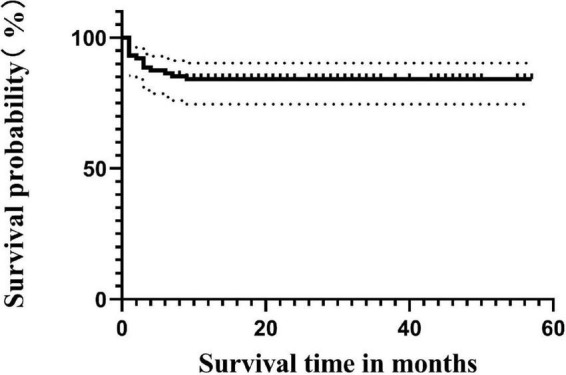
Survival curves up to 48 months.

**FIGURE 2:**
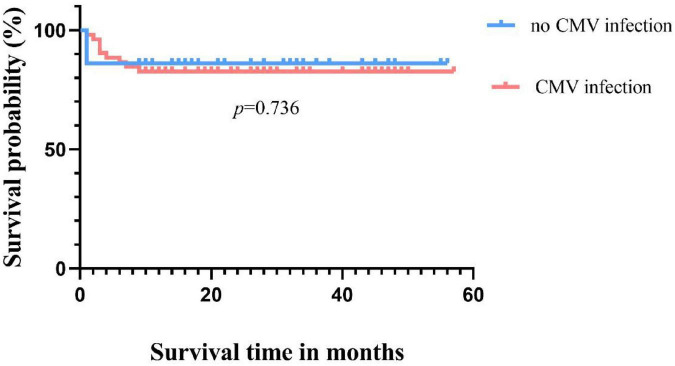
Overall survival with and without CMV infection.

Until the follow-up endpoint, a total of 14 patients had died, with the specific causes as follows: 4 died from sepsis, 4 from severe pneumonia, 1 from cerebrovascular accident, 2 from aGVHD, 2 from post-transplant lymphoproliferative disorder (PTLD), and 1 from acute heart failure. The 4-year OS rate for all 88 patients was 84.1% (Figure 1). The survival rate for the non-CMV infection group was 86.1% (31/36), and for the CMV infection group, it was 82.7% (43/52), with no statistically significant difference in survival time between the two groups (*p* = 0.736) (Figure 2).

**FIGURE 3:**
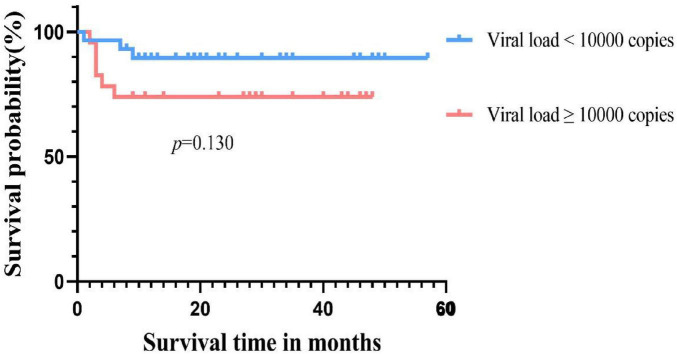
Overall survival between with low viral load group and the high viral load group.

Among the 52 patients with CMV infection, the OS rate was 50% (6/12) in the RCI group and 92.5% (37/40) in the non-RCI group, with a statistically significant difference in survival outcomes between the two groups (*p* = 0.000). For the group with the highest viral load above 1.0 × 10^4^ copies/ml, the survival rate was 73.9%, while for the group with a load below 1.0 × 10^4^ copies/ml, the survival rate was 89.5%, with no statistically significant difference in survival time between the two groups (*p* = 0.130) (Figure 3).

### 3.5 Analysis of factors influencing survival

A univariate and multivariate Cox regression analysis was conducted to explore potential factors influencing the survival time of patients post-transplantation. The results are presented in Table 3. Univariate analysis indicated that recipient age >40 years (*p* = 0.017), unassisted infusion of mesenchymal stem cells (*p* = 0.019), neutrophil engraftment beyond 28 days (*p* = 0.005), platelet engraftment beyond 28 days (*p* = 0.000), and non-EBV infection (*p* = 0.017) were risk factors affecting patient survival. Multivariate Cox regression analysis identified platelet engraftment beyond 28 days (HR = 0.132, 95% CI 0.036∼0.481, *p* = 0.002) as an independent risk factor influencing the survival time of patients.

**TABLE 3:** Univariate and multivariate analysis of factors influencing overall survival after allogeneic hematopoietic stem cell transplantation.

Factors	Univariate analysis			Multivariate analysis		
	**HR**	**95% CI**	***p*-Value**	**HR**	**95% CI**	***p*-Value**
Age	3.280	1.148∼9.371	0.017	1.273	0.359∼4.520	0.709
Sex	0.975	0.342∼2.781	0.962	–	–	–
Donor sex	0.337	0.094∼1.207	0.075	–	–	–
Donor/recipient sex combination	3.575	0.468∼27.335	0.183	–	–	–
Diagnosis	0.934	0.260∼3.347	0.915	–	–	–
The blood type of the donor and the recipient	1.144	0.383∼3.414	0.806	–	–	–
Stem cell source	0.459	0.128∼1.645	0.213	–	–	–
Conditioning regimen	1.214	0.339∼4.351	0.762	–	–	–
UC-BSC assisted reinfusion	0.591	0.205∼1.703	0.317	–	–	–
MSC assisted reinfusion	0.277	0.087∼0.885	0.019	0.320	0.092∼1.116	0.074
NE 28-day engraftment	0.194	0.054∼0.703	0.005	1.101	0.251∼4.821	0.899
PLT 28-day engraftment	0.105	0.033∼0.336	0.000	0.132	0.036∼0.481	0.002
aGVHD	1.185	0.416∼3.379	0.747	–	–	–
cGVHD	0.602	0.168∼2.159	0.424	–	–	–
EBV infection	0.290	0.097∼0.867	0.017	1.393	0.345∼5.626	0.642
CMV infection	1.203	0.403∼3.590	0.736	–	–	–

SAA, severe aplastic anemia; NSAA, non-severe aplastic anemia; Bu, busulfan; Cy, cyclophosphamide; FCA, fludarabine, cyclophosphamide, antithymocyte globulin; NE, neutrophils; PLT, platelet; aGVHD, acute graft-versus-host disease; cGVHD, chronic graft-versus-host; EBV, Epstein-Barr virus; CMV, cytomegalovirus; –, multivariate analyses were not included.

## 4 Discussion

Following the initial infection, CMV establishes a lifelong latent infection in the host under the control of the immune response. Reactivation of CMV is a common event in recipients of allo-HSCT. In this study, 59.1% (52/88) of patients experienced CMV infection during the postoperative follow-up period, with 23.1% (12/52) of CMV-infected patients developing RCI. The incidence of CMV infection and RCI in this study is similar to previous reports (4, 5). Without the use of letermovir, exploring risk factors for CMV infection may provide new insights into treatment strategies.

In previous studies, recipient seropositive status, graft source, transplantation type, HLA compatibility, and GVHD have been identified as common risk factors for CMV infection (20–22). We observed that among the 26 patients who developed cGVHD, there was an 81% incidence of CMV infection. This observation leads us to infer that cGVHD is a significant risk factor for CMV infection, and our study confirms this hypothesis. Our data indicate that cGVHD is an independent risk factor for CMV infection. This finding is not entirely consistent with previous research results (22). On the one hand, there may be a reciprocal interaction between CMV virus and cGVHD. This could be related to the type of disease, as the CMV virus is more likely to infect when T cells are deficient or impaired. In the case of AA transplantation, long-term use of immunosuppressive agents is required, leading to a slower immune reconstitution compared to other hematological malignancies after transplantation, thus providing opportunities for extended periods of immune reconstitution recovery, which may increase the risk of infection. On the other hand, the EBV may contribute to CMV infection by influencing aGVHD and cGVHD.

The main pathophysiological process of cGVHD is immune-mediated inflammatory response. Chronic inflammation causes thymus damage and B cell and T cell immune disorder, which eventually leads to tissue fibrosis. T lymphocytes can cause tissue damage and fibrosis through direct cytolysis and cytokine secretion (23, 24), especially CD4+ T lymphocytes, whose interaction with B cells promotes B cell differentiation and the production of autoantibodies. These include antibodies against cytoskeletal intermediate filaments, cytoplasmic squamous epithelial cells, and nucleolar B23, These antibodies participate in inflammation and activate signal transduction pathways, leading to increased expression of type I collagen genes, promoting fibroblast activation, and inducing typical cGVHD clinical symptoms such as skin sclerosis and pulmonary fibrosis. In addition, T cell subsets play a crucial role in the immune regulation of cGVHD. Activation of the NOTCH2 signaling pathway in B cells has a profound effect on T cell subsets, including helper T cells (Th) and regulatory T cells (Treg) (25–27). This results in delayed immune reconstitution after allo-HSCT, increased risk of death and cGVHD, and increased risk of CMV reactivation. In addition, since patients with AA use immunosuppressants longer after transplantation than those with other hematological malignancies, it is more likely to cause delayed immune reconstitution after transplantation.

There is limited research on the correlation between cGVHD and CMV infection, but the relationship between cGVHD and CMV infection is not absent. Olkinuora et al. (28) found that mild aGVHD and cGVHD can promote the recovery of cellular and humoral immunity, while moderate to severe cGVHD hurts immune recovery after transplantation. Furthermore, active CMV infection can contribute to the occurrence and exacerbation of cGVHD by increasing levels of IL-2 and tumor necrosis factor-alpha in peripheral blood (29).

Previous research results indicate that the influence of CMV infection on aGVHD is affirmative (30, 31). The study by Styczynski (32) indicates that the incidence of CMV infection in patients with aGVHD is almost twice that of patients without aGVHD [*p* < 0.0001, 60.1% (885/1,472) vs. 32.1% (892/2,780)]. Cantoni et al. (33) found that GVHD and its treatment can induce CMV replication, and CMV replication increases the risk of GVHD occurrence (34, 35). It is noteworthy that in our study, there was no significant difference in the incidence of CMV infection between patients with and without aGVHD.

As is well-known, EBV, as one of the common viral infections after allo-HSCT, is also a routine monitoring indicator. Previous studies have suggested that EBV infection increases the incidence of II–IV degree aGVHD and cGVHD (34). Since CMV infection is influenced by aGVHD, EBV infection may indirectly affect CMV infection by influencing post-transplant immune reconstitution. There is a complex interrelationship between CMV infection, EBV infection, and the occurrence of GVHD. However, current research on the impact of EBV infection on CMV infection is limited, and the relationship among these three factors is not yet clear. Interestingly, in this study, univariate analysis found an association between the occurrence of CMV infection and cGVHD as well as EBV infection, which warrants further in-depth investigation.

Cytomegalovirus infection has a significant impact on the prognosis of patients, especially with a higher mortality rate in CMV disease and RCI, significantly affecting patient survival (36, 37). In our study, although there was no significant difference in survival rates between CMV-infected and uninfected patients (82.7% vs. 86.1%, *p* = 0.736), the occurrence of RCI was associated with shorter OS compared to the non-RCI group (50% vs. 96.6%, *p* = 0.000), consistent with previous reports (4, 5). The direct and indirect effects of CMV in this study may negatively influence patient prognosis in different ways. On the other hand, consistent with Green et al., a higher CMV viral load after transplantation was associated with an increased risk of death (adjusted hazard ratio [HR] 19.8, 95% CI 9.6–41.1) (38). However, in our cohort, the peak viral load of CMV reactivation in transplant recipients was used as a qualitative parameter. The CMV-infected patients were divided into two groups based on the highest viral load, with a threshold of 1.0 × 10^4^ copies/ml. Regarding the final survival rate, no significant difference was observed between the low viral load group and the high viral load group (89.5% vs. 73.9%, *p* = 0.130). This may be influenced by sample size and other factors. However, under the same treatment, the low viral load group had a higher rate of turning negative compared to the high viral load group (96.6% vs. 73.9%, *p* = 0.035). This suggests that patients with lower viral loads are more likely to turn negative and have a higher survival rate under the same treatment regimen, thereby improving the prognosis. This also emphasizes the importance of closely monitoring CMV, and with the advent and clinical application of letermovir (39). These patients may benefit from letermovir. Therefore, early intervention, especially after discontinuing prophylaxis, may be considered if necessary. On the other hand, the quantitative definition of pre-transplant CMV serostatus, rather than qualitative, influences the 3-year survival rate after allo-HSCT. This provides new insights into the negative prognostic impact of CMV on transplant recipients (35). However, the lack of pre-transplant serostatus in some patients in this cohort is a limitation of this study. The CMV seropositivity rate of Chinese HSCT patients is as high as 80%–93.7%, which is much higher than that in European and American countries. Therefore, although some patients in this study lack serological status, we can still speculate that they are at risk of CMV reactivation.

Furthermore, we attempted survival analysis, indicating that CMV infection was not statistically significant. Factors such as hematopoietic reconstruction and age may influence patient OS, and failure of platelet engraftment within 28 days (*p* = 0.002) emerged as an independent risk factor affecting patient OS. This differs from previous studies reporting CMV infection as an independent prognostic factor. It is considered that this discrepancy may be due to the combined influence of other factors, and further studies with an expanded sample size are needed to validate these findings.

## 5 Conclusion

In summary, further emphasis on monitoring CMV-DNA in transplant recipients is warranted, particularly in patients developing cGVHD, necessitating proactive prevention of CMV infection. High viral load patients should receive more aggressive treatment to prevent RCI occurrence, early combination therapy when necessary. Once CMV infection progresses to RCI, the prognosis is poor. Actively promoting hematopoietic reconstruction, preventing the occurrence of CMV infection, and controlling the development of CMV infection can lead to improved survival in AA patients undergoing Haplo-HSCT. In addition, we should pay close attention to the level of T lymphocyte subsets to evaluate cellular immune reconstitution, and rationally adjust immunosuppressants to further reduce CMV reactivation. For patients who use letermovir to prevent CMV infection, we can also further study its effect on the level of T lymphocyte subsets and cellular immune reconstitution.

## Data Availability

The raw data supporting the conclusions of this article will be made available by the authors, without undue reservation.

## Supplementary material

The supplementary material is the original data table.
